# An ultra-deep TSV technique enabled by the dual catalysis-based electroless plating of combined barrier and seed layers

**DOI:** 10.1038/s41378-024-00713-5

**Published:** 2024-06-11

**Authors:** Yuwen Su, Yingtao Ding, Lei Xiao, Ziyue Zhang, Yangyang Yan, Zhifang Liu, Zhiming Chen, Huikai Xie

**Affiliations:** 1 School of Integrated Circuits and Electronics, Beijing Institute of Technology, Beijing, China; 2 Chongqing Institute of Microelectronics and Microsystems, Beijing Institute of Technology, Chongqing, China

**Keywords:** Electrical and electronic engineering, Engineering

## Abstract

Silicon interposers embedded with ultra-deep through-silicon vias (TSVs) are in great demand for the heterogeneous integration and packaging of opto-electronic chiplets and microelectromechanical systems (MEMS) devices. Considering the cost-effective and reliable manufacturing of ultra-deep TSVs, the formation of continuous barrier and seed layers remains a crucial challenge to solve. Herein, we present a novel dual catalysis-based electroless plating (ELP) technique by tailoring polyimide (PI) liner surfaces to fabricate dense combined Ni barrier/seed layers in ultra-deep TSVs. In additional to the conventional acid catalysis procedure, a prior catalytic step in an alkaline environment is proposed to hydrolyze the PI surface into a polyamide acid (PAA) interfacial layer, resulting in additional catalysts and the formation of a dense Ni layer that can function as both a barrier layer and a seed layer, particularly at the bottom of the deep TSV. TSVs with depths larger than 500 μm and no voids are successfully fabricated in this study. The fabrication process involves low costs and temperatures. For a fabricated 530-μm-deep TSV with a diameter of 70 μm, the measured depletion capacitance and leakage current are approximately 1.3 pF and 1.7 pA at 20 V, respectively, indicating good electrical properties. The proposed fabrication strategy can provide a cost-effective and feasible solution to the challenge of manufacturing ultra-deep TSVs for modern 3D heterogeneous integration and packaging applications.

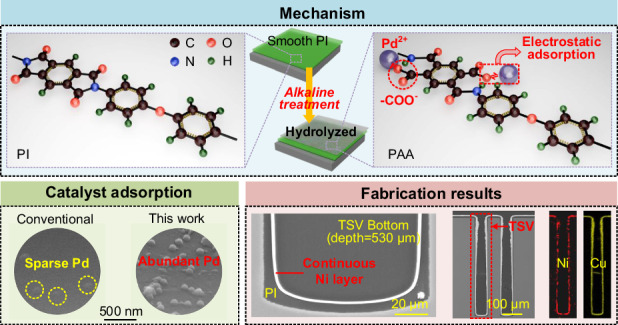

## Introduction

With the rapid development of information science and technology, the demands for both high-performance integrated circuits (ICs) and multi-functional integrated systems are continuously increasing^1^^,^^2^. By introducing electrical interconnects in the vertical orientation, 2.5D/3D integration technologies based on through-silicon vias (TSVs) can be implemented to realize the heterogeneous integration of multiple chips with different functionalities and material compositions through various processes on a single packaging substrate with very short interconnects^3–6^. Therefore, TSV-based 2.5D/3D integration technologies can not only reduce the complexity of the system but also enable multi-functional integration characteristics and high bandwidths; thus, these technologies have been regarded as highly effective and promising solutions for extending and even surpassing Moore’s Law^7–9^.

In most cases, advancements in TSV technology are focused on increasing the TSV density with small TSV depths (<100 μm)^10–12^. Recently, driven by the need for the heterogeneous integration and packaging of chiplets with various opto-electronic functionalities and movable microelectromechanical systems (MEMS) structures, TSVs with large depths (>400 μm) are highly desirable for ensuring optical flatness and mechanical robustness characteristics using thick interposer substrates^13–16^. While TSV technology has been extensively studied in previous decades^17–19^, the manufacturing of ultra-deep TSVs still requires further investigation because increasing TSV depth can greatly increase the difficulties and complexities of fabrication. Specifically, the formation of continuous and dense barrier and seed layers on the sidewall surfaces and bottoms of ultra-deep TSVs are very challenging in terms of continuity and processing costs.

As shown in Fig. S1a, the functional layers on the sidewall of a TSV consist of a dielectric liner (mostly SiO_2_ or a polymer), a barrier (usually a metal such as TiN, TiW, and TaN), and a seed layer (normally Cu). The dielectric liner can be readily deposited by processes such as the thermal oxidation or spin-coating of a polymer. However, the deposition of barrier and seed layers is more complicated. Physical vapor deposition (PVD)^20^, atomic layer deposition (ALD)^21,22^, and electroless plating (ELP)^23–26^ are usually utilized to deposit barrier and seed layers separately. Due to the poor step coverage of PVD, a relatively thick layer must be deposited to ensure its continuity at the bottom of the TSV (e.g., 800 nm at the top of the via is needed to achieve 30 nm at the bottom of the via)^27,28^. ALD is excellent for forming conformal layers in TSVs^22,23^, but this method suffers from a low deposition rate, which is typically lower than 1 nm per minute, leading to a high cost^29^. Recently, electroless plating (ELP) has emerged as a promising alternative due to its low cost, convenient operation, and conformal coating characteristics^23–26,30^. In previous decades, efforts have been made to enhance the formation of catalysts (typically Pd) on the via sidewall, which is crucial for ensuring the ELP of metal layers. Since 2014, it has been reported that depositing an additional metal interlayer such as Ru^24^, Co^25^, and Cu^23,31^ before performing ELP can facilitate the adsorption of Pd catalysts, thus achieving the continuity of Cu seed layers in TSVs with depths up to about 141 μm. In 2022, Xiao et al.^32^ presented the formation of dense Cu seed layers in ultra-deep TSVs utilizing ultrasound-assisted ELP without an additional interlayer. However, a barrier layer (TiN) is still needed between the liner and seed layer, which is typically realized by performing an expensive and time-consuming ALD procedure.

Given the good barrier characteristics of Ni^33–35^, electroless Ni plating for a combined barrier/seed layer (Fig. S1b) can be implemented to further simplify the fabrication flow. Nevertheless, it is rather difficult to directly bind Pd nanoparticles on the surfaces of polymer liners, such as polyimide (PI)^36,37^, which have been widely used as alternatives to conventional SiO_2_ liners^38–40^ and as functional layers in advanced MEMS and electronic devices^41–43^. This issue is intensified for high-aspect-ratio or ultra-deep TSVs due to the low solute concentrations at their bottom ends. Hu et al.^44^ reported enhanced adsorption of Pd catalysts on polymer liners treated in sodium borohydride (NaBH_4_) solution and achieved a high aspect ratio of 10, with a TSV depth that remained in the tens of microns. Considering that ELP is a wet process that relies on a series of interfacial chemical reactions in a specific order and weakens at the TSV bottoms due to the inadequate diffusion and transport capabilities of reactant solutes, the process difficulty dramatically increases for ultra-deep TSVs. Moreover, the interfacial modification methods for facilitating the adsorption of Pd nanoparticles on liner surfaces through electrochemical techniques provide inspiration for this research. In summary, it is imperative and beneficial to enable the fabrication of barrier/seed layers without using ALD, PVD, or chemical vapor deposition (CVD), particularly in ultra-deep TSVs.

In this study, a novel dual catalysis-based Ni ELP technique is presented for forming continuous combined barrier/seed layers on PI liner in ultra-deep TSVs with a single process. With the vacuum-assisted spin-coating of the PI liner, the electroplating of the annular Cu conductor, and the vacuum-assisted filling of the benzocyclobutene (BCB) central filler, a low-cost and low-temperature fabrication scheme is demonstrated, and ultra-deep TSVs are successfully fabricated. Finally, the electrical properties, including the capacitance–voltage (C–V) and leakage current, are evaluated. This work can contribute to the development and implementation of the heterogeneous integration and packaging of multi-functional chiplets by Si interposers with embedded ultra-deep TSVs.

## Materials and methods

### Dual catalysis-based ultrasound-assisted ELP of Ni

Within the fabrication process of ultra-deep TSVs, a crucial yet challenging step is the formation of continuous and dense barrier and seed layers. In this work, a novel technique labeled the dual catalysis-based ultrasound-assisted ELP is developed for depositing Ni layers in ultra-deep TSVs, which function as combined barrier/seed layers.

**Fig. 1:**
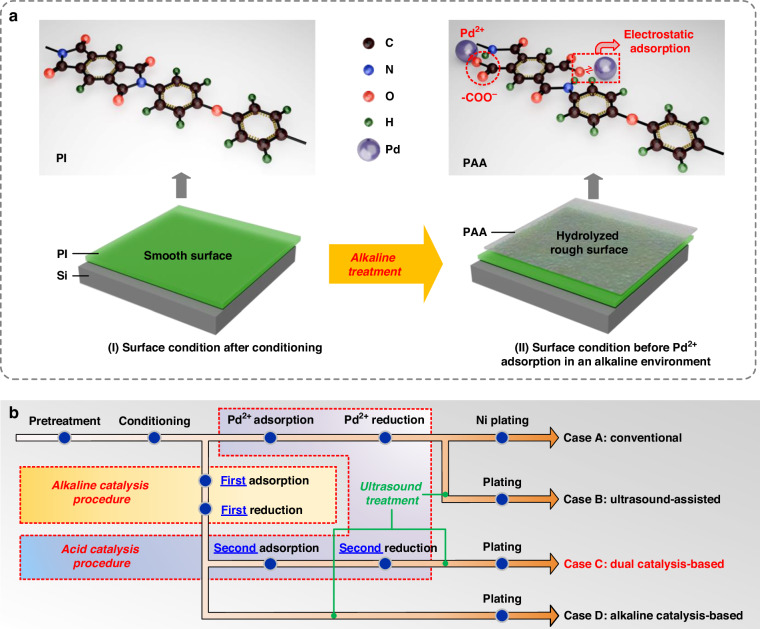
Key modification of the dual catalysis-based ELP and conceptual design processes of the contrast experiments **a** Schematic diagram of the effect of alkaline treatment on the surface of PI liner during alkaline catalysis. **b** Schematic diagram of the four cases for the ELP of Ni. Case A was the conventional ELP; Case B was the ultrasound-assisted ELP where ultrasound treatment was introduced to facilitate the Ni plating; Case C was the proposed dual catalysis-based ELP involving an additional catalysis procedure in an alkaline environment; and Case D was the ELP with only one catalysis procedure in an alkaline environment serving as a comparison

In this technique, an additional catalytic step in an alkaline environment was introduced before the conventional acid catalysis procedure to modify the PI surface. This step involved the hydrolysis of the imide structure of the surface PI liner into an amide structure, exposing -COO^−^ groups that easily attracted metal cations through electrostatic adsorption^36,44,45^. This phenomenon resulted in enhanced Pd^2+^ adsorption on the formed polyamide acid (PAA) interfacial layer through electrostatic adsorption, as shown in Fig. 1a. The increased Pd catalysts contributed to the subsequent formation of the continuous Ni layer, which held significant importance as it ensured the high-quality electroplating of the Cu conductor and concurrently protected the PI liner from potential Cu diffusion. Therefore, a series of comprehensive experiments focusing on the ELP of Ni was undertaken to validate the feasibility of this critical stage in the TSV fabrication flow process.

As shown in Fig. 1b, four distinct cases were experimentally investigated, including conventional ELP (Case A), ultrasound-assisted ELP (Case B), dual catalysis-based ultrasound-assisted ELP (Case C), and alkaline catalysis-based ultrasound-assisted ELP (Case D).

In Case A, the conventional ELP process was composed of pretreatment, conditioning, adsorption of Pd ions, and reduction of Pd and Ni ions, as shown in Fig. 1b. An off-the-shelf conditioning ELP solution (Melplate PC 6160, Meltex Inc.) was used in this work. The wafer initially underwent a pre-treatment procedure. It was first immersed in deionized (DI) water and then placed in a vacuum chamber to ensure the wetting of the entire via sidewall. Then, the wafer was transferred into a conditioning solution for a 5-minute water-bath heating process at 60 °C to clean its surface. After the conditioning step, the adsorption of Pd^2+^ occurred in the activator solution at room temperature for 20 min, which was an acid solution containing [PdCl_4_]^2−^. Then, the reduction of the adsorbed Pd^2+^ and Ni^+^ was carried out in the reducer solution at 80 °C with NaH_2_PO_2_. The reduction time was set to 90 min.

In Case B, ultrasound treatment was incorporated into Case A while reducing Pd and Ni ions. The ultrasound frequency was set to 25 kHz, and the ultrasound power and pulse duty cycle were set to 300 W and 1 s/10 s, respectively, following experimental parametric analyses.

Compared to Case B, the proposed dual catalysis-based ELP technique in Case C was critically innovated by introducing an additional catalysis procedure in an alkaline environment before performing the conventional acid catalysis procedure, as shown in Fig. 1b. During the first alkaline catalysis procedure, the wafer was initially immersed in the alkaline activator solution (pH>11) containing PdSO_4_ for the first adsorption of Pd^2+^ at 40 °C for 20 min. Subsequently, the wafer was immersed in another alkaline reducer solution (pH>8) containing dimethylamineborane (DMAB) for the first reduction of Pd^2+^ at 35 °C for 20 min. The process then proceeded with the second acid catalysis procedure including the second adsorption of Pd^2+^ and the second reduction of the adsorbed Pd^2+^ and Ni^+^. The temperatures used in the alkaline catalysis procedure were empirical parameters selected from the vendor (Atotech Ltd.) to balance the activities of the reactants and the lifetimes of the solutions.

Moreover, serving as a comparison to Case C, Case D reserved the first catalysis procedure in the alkaline environment while omitting the second procedure in the acid environment.

### Vacuum-assisted spin-coating of PI liner

In this work, the PI liner was fabricated using a vacuum-assisted spin-coating technique^39^. A PI solution consisting of PI (PI-5J, YiDun New Material Co., Ltd., China) and an organic solvent dimethylacetamine (DMAc) with a 3:1 volume ratio was employed. After etching blind vias by standard deep reactive ion etching (DRIE) of Bosch process, the PI solution was dispensed onto the surface of a silicon wafer. The wafer was immediately moved into a vacuum chamber for a 3-minute vacuum treatment. During this process, the trapped air in the deep vias was evacuated in the vacuum chamber, so the PI solution filled the vias. Subsequently, the wafer underwent high-speed spinning at 3000 rpm for 40 s on a spin coater. The spinning expelled most of the PI solution in the vias, leaving behind thin PI layers on the sidewalls of the vias. Finally, the PI layer was cured in a N_2_ environment at 240 °C with a duration of 4 h, resulting in conformal PI liners in deep vias.

### Electroplating of Cu and vacuum-assisted filling of BCB

After the formation of the Ni barrier/seed layer, Cu electroplating was carried out to deposit annular Cu conductors, utilizing commercial solutions with careful consideration given to the extensive depths of the TSVs. To prevent sealing, the current density was maintained at a relatively low value of 0.1 A/dm^2^. The electroplating duration was 7 h.

The BCB central fillers were fabricated through vacuum-assisted filling. Initially, the adhesion promoter AP3000 (Dow Chemistry Co.) was filled into the central trenches through a 2-minute vacuum treatment. Then, the sample was spun on a spin-coater at 500 rpm for 6 s and 3000 rpm for 40 s, followed by baking at 95 °C for 10 min. Thus, a thin adhesion promoter layer was formed. In the second step, the central trenches were completely filled with BCB in a vacuum chamber, and the sample was spun at 500 rpm for 6 s and 1000 rpm for 40 s to thin and smooth the initially thick BCB layer. Finally, the BCB was cured in an ambient N_2_ atmosphere at 250 °C for 80 min.

### Experimental characterization

The characterization of key steps during fabrication was performed by taking cross-sectional micrographs using scanning electron microscopy (SEM; FEI QUANTA 450). Elemental mapping after the deposition of Ni and completion of the TSV fabrication was conducted through energy-dispersive X-ray spectrometry (EDS), employing an EDAX detector integrated into the SEM system. Additionally, X-ray photoelectron spectroscopy (XPS; ULVAC PHI QUANTERA II) under Al Kα (1486.6 eV) radiation was utilized to assess the chemical structure and component of the sample surface before and after performing the catalyst adsorption steps during ELP. Spectral energies were calibrated with the binding energy of C 1 s being set to 284.8 eV. Furthermore, electrical characterizations of the fabricated TSVs were carried out at room temperature using a Keysight B1500A semiconductor parameter analyzer with a Cascade Summit 11000 probe station. The test assembly is shown in Fig. S2.

## Results and discussion

### Ni deposition under different ELP cases

**Fig. 2:**
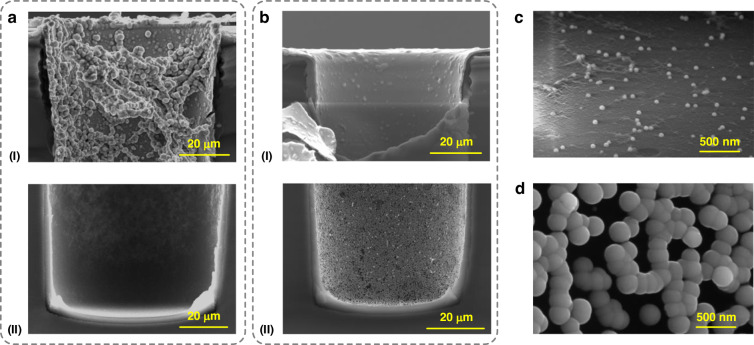
Deposition results of Cases A and B **a** SEM images of the Ni ELP results of Case A at the (I) via top and (II) via bottom. **b** Ni ELP results of Case B after performing ultrasound treatment with ultrasound power and pulse duty cycle values of 300 W and 1 s/10 s, respectively. **c**, **d** Magnified images of the via bottoms for Case A and B, respectively, showing the distributions and morphologies of the deposited Ni particles

In conventional ELP (Case A), both the adsorption of Pd ions and the reduction of Pd and Ni ions occurred in an acid environment. However, ultra-deep TSVs with PI liners encountered two major issues: 1) the accumulation of gas bubbles during reduction impeded solute diffusion into the vias, particularly at their top ends, and 2) the limited adsorption of Pd ions on the PI interface in an acid environment, which resulted in sparse Pd particles, especially at the bottom ends of the vias. These issues led to the clustering of Ni particles near the via tops and scattered Ni particles at the via bottoms, as shown in Fig. 2a.

In Case B, the cavitation effect of ultrasound treatment helped to disperse the gas bubbles, which were mainly generated during Ni plating, preventing their aggregation at the via top and facilitating solvent diffusion into deep vias, as shown in Fig. S3. Notably, ultrasound treatment was not applied during alkaline catalysis since the number of Pd catalysts was low. The ultrasound treatment was applied during the alkaline catalysis procedures in Case C and Case D, but there were no obvious differences in the deposition results. Since the adhesion between the Pd catalysts and the PI liner was not strong, we did not suggest the application of ultrasound treatment during alkaline catalysis. The effects of ultrasound power and pulse duty cycle on Ni deposition were evaluated, and the results are shown in Figs. S4 and 2b. Figure S4a–c shows that a high ultrasound power resulted in an increasingly uniform Ni layer at the via top. However, at a low pulse duty cycle, the Ni layer at the via bottom remained scarce. The enhanced uniformity at the via top with a high ultrasound power was attributed to the efficient breaking of gas bubbles accumulated in this region. Nevertheless, due to the low pulse duty cycle, solute transportation into the deep via bottom was hindered, limiting the improvement in the Ni layer in this region. Figures S4d and 2b show deposition results with high pulse duty cycles while maintaining the ultrasound power at 300 W. A relatively high pulse duty cycle further improved the smoothness of the Ni layer at the via top and increased the number and density of Ni particles at the via bottom. Notably, both ultrasound power and pulse duty cycle could not be overly high to prevent the exfoliation of deposited Ni from the PI surface.

Consequently, the optimal conditions for Case B were set to 300 W for ultrasound power and 1 s/10 s for pulse duty cycle. As shown in Fig. 2b, the deposited Ni in Case B was conformal and formed a continuous layer at the via top. However, despite the increased Ni density at the via bottom shown in Fig. 2b(II) compared to that of Case A shown in Fig. 2a(II), a continuous layer was not created due to the poor growth of Pd catalysts. Figure 2c, d show the enlarged via bottom areas for Case A and Case B, respectively, providing detailed views of the distributions and morphologies of the deposited Ni particles and emphasizing the improvements achieved by performing ultrasound treatment in Case B.

**Fig. 3:**
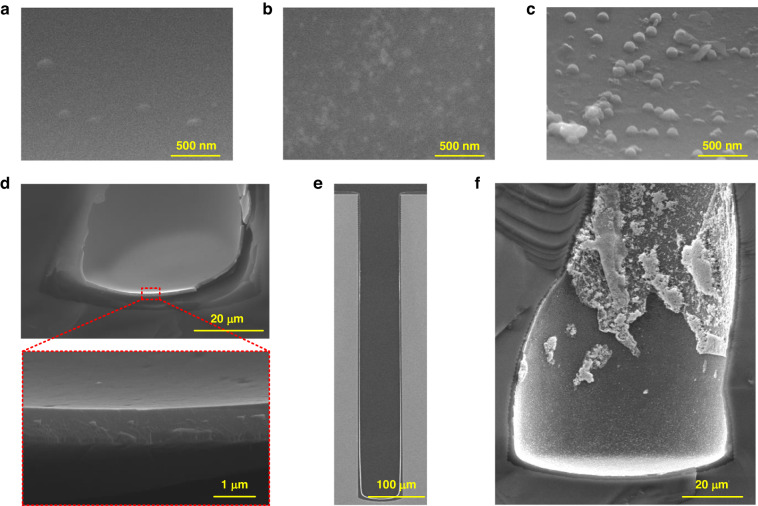
SEM images of the deposited Pd catalysts and the resulting Ni layers at the via bottoms for case B, case C, and case D Formed Pd catalysts at the via bottom of **a** Case B, **b** Case C after the first Pd^2+^ reduction, and **c** Case C after the second Pd^2+^ reduction. **d**, **e** ELP results of Ni in Case C. The inset of **d** is an enlarged image of the via bottom, revealing the dense Ni layer. The SEM image in **e** is obtained after metallographic polishing, indicating the continuity of the fabricated Ni layer. **f** Via bottom of Case D after Ni^+^ reduction, showing the irregular growth of Ni

Figure 3a, b show the formed Pd catalysts at the TSV bottoms after reducing the Pd ions in acid and alkaline environments, respectively. In Fig. 3a (Case B), only a few Pd particles were observed, while Fig. 3b (after the first Pd^2+^ reduction for Case C) shows abundant tiny Pd particles. This enhancement was attributed to the modification of the PI liner surface by alkaline treatment, as shown in Fig. 1a. Furthermore, since the reduction of Ni ions must occur in an acid environment, catalyst deposition in an acid environment was retained as the second catalyst deposition in Case C. Figure 3c shows the Pd catalysts at the via bottoms in Case C after performing dual catalysis. Compared to Fig. 3b, the Pd particles in Fig. 3c appeared even larger in both quantity and size, as the second catalyst deposition step in the acid environment contributed additional catalysts.

As a result, the realization of abundant catalysts in the proposed dual catalysis-based ELP technique facilitated the formation of continuous Ni layers in ultra-deep TSVs with diameters of 70 μm and depths of 530 μm, even at the deep via bottom, as shown in Fig. 3d. The SEM images in Fig. 3e obtained after metallographic polishing confirm the good continuity of the deposited Ni layer. Figures S5a–c show the enlarged views of the via top, middle, bottom of the TSV in Fig. 3e (this TSV was located at the 1/2 radius region of the wafer). In addition, Fig. S5d shows the EDS image for the Ni shown in Fig. S5c. Figures S5d, e present two other TSVs for Case C from different regions of the wafer. The thicknesses of the deposited Ni layers in the three TSVs in different areas are plotted in Fig. S5g. The good continuity of the deposited Ni layers with a minimum thickness of ~100 nm and an especially dense bottom highlighted the feasibility of the proposed dual catalysis-based ELP technique and facilitated the fabrication of ultra-deep TSVs. The Ni layers in TSVs from different regions of the wafer were highly consistent, proving the good yield of the proposed technique. In contrast, Fig. 3f shows the deposited Ni in Case D which had only one catalysis procedure in the alkaline environment. Despite the abundance of formed Pd catalysts after the first Pd^2+^ reduction in an alkaline environment, as shown in Fig. 3b, the deposited Ni could not form a continuous layer due to the abnormal Ni ion reduction characteristics in an alkaline environment. Therefore, the second catalyst deposition step in an acid environment (Case C) was considered necessary for the success of the dual catalysis-based ELP.

### Surface characterizations and mechanism analyses

**Fig. 4:**
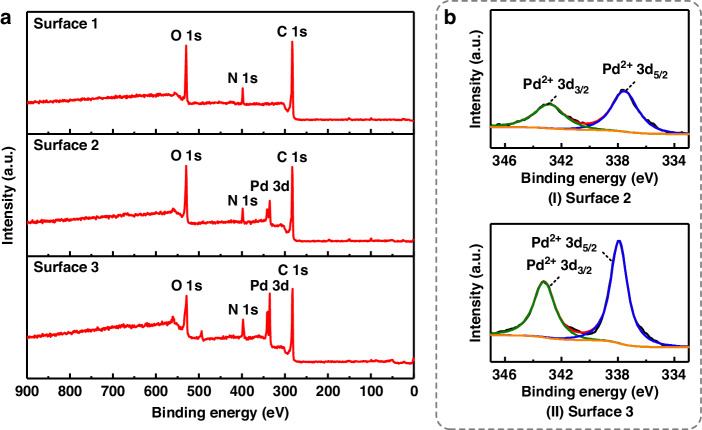
XPS spectra of the three surfaces **a** Wide scan. **b** Pd 3d. The Pd 3d spectrum of Surface 1 is omitted as Pd element has not been involved at this stage

To elucidate the underlying mechanisms that lead to the enhancement of catalyst adsorption on the PI liner interface by introducing catalysis in an alkaline environment, three samples with fabricated PI liners were characterized by XPS after performing different steps during ELP. The analyzed surfaces included the conditioning step (Surface 1), Pd^2+^ adsorption in an acid environment of Case A/B (Surface 2), and Pd^2+^ adsorption in an alkaline environment of Case C/D (Surface 3). Figure 4 shows the XPS wide scan spectra and the high-resolution Pd 3d spectra for these surfaces. As shown in Fig. 4a, Pd 3d peaks were observed in the wide scan spectra of Surfaces 2 and 3, indicating the existence of adsorbed Pd ions. Notably, the Pd 3d peak intensities of Surface 3 were much larger than those of Surface 2, as confirmed by the high-resolution Pd 3d spectra shown in Fig. 4b, where the intensities of both the Pd^2+^ 3d_3/2_ (~343.1 eV) and Pd^2+^ 3d_5/2_ (~337.8 eV) peaks were stronger for Surface 3 than for Surface 2.

**Fig. 5:**
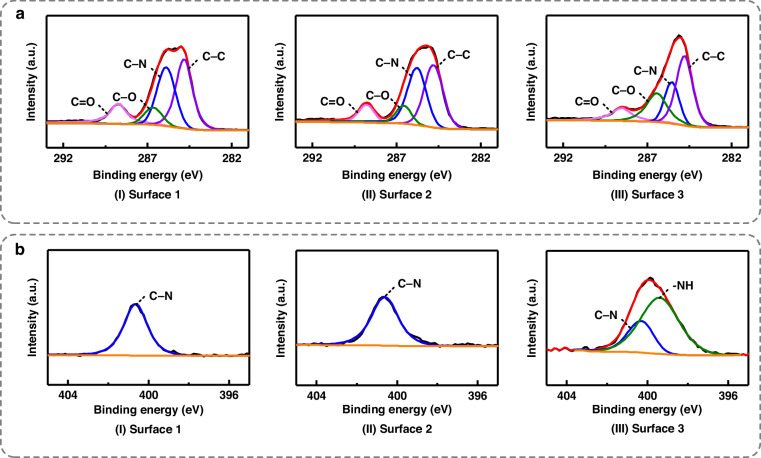
XPS spectra of the three surfaces **a** C 1 s. **b** N 1 s

Figure 5 shows the C 1s and N 1s XPS spectra of the three surfaces. For Surface 1, a typical PI structure was observed. The four peaks in the C 1 s spectrum in Fig. 5a(I), at 284.8 eV, 285.8 eV, 286.7 eV, and 288.8 eV were attributed to C-C, C-N, C-O, and C = O bonds, respectively^46^. The N 1 s spectrum in Fig. 5b(I) shows a single peak corresponding to the C-N bonds at 400.6 eV^46^. Surface 2 exhibited no significant changes in the morphologies of the component peaks in the C 1 s and N 1 s spectra, indicating a lack of obvious alternations in the chemical structure and component of the PI surface after Pd^2+^ adsorption in an acid environment. In contrast, Surface 3 showed notable variations in the XPS spectra. On the one hand, the peak intensities of the C-N bonds decreased, and the C-O bonds in the C 1 s spectra were enhanced, as shown in Fig. 5a(II) and 5a(III). On the other hand, the N 1 s spectra showed decreases in the intensities of the C-N bonds with the appearance of a new component peak at approximately 399.5 eV, corresponding to the presence of -NH groups, as shown in Fig. 5b(II) and 5b(III)^44^. It was inferred that the imide structure of the PI surface exposed to the alkaline solution was hydrolyzed to form the amide structure^36^, leading to changes in the C-O and C-N bonds and the appearance of the -NH groups. The generated -COO^−^ groups attracted the Pd^2+^ ions through electrostatic adsorption, increasing the intensities of the Pd 3d peaks. The Pd percentage of Surface 3 increased from 1.66% to 3.28%, compared to Surface 2. These results were in agreement with the analyses and fabrication outcomes, providing insights into the improvements induced by the additional catalysis procedure performed in an alkaline environment.

### Fabrication and electrical characterization methods

**Fig. 6:**
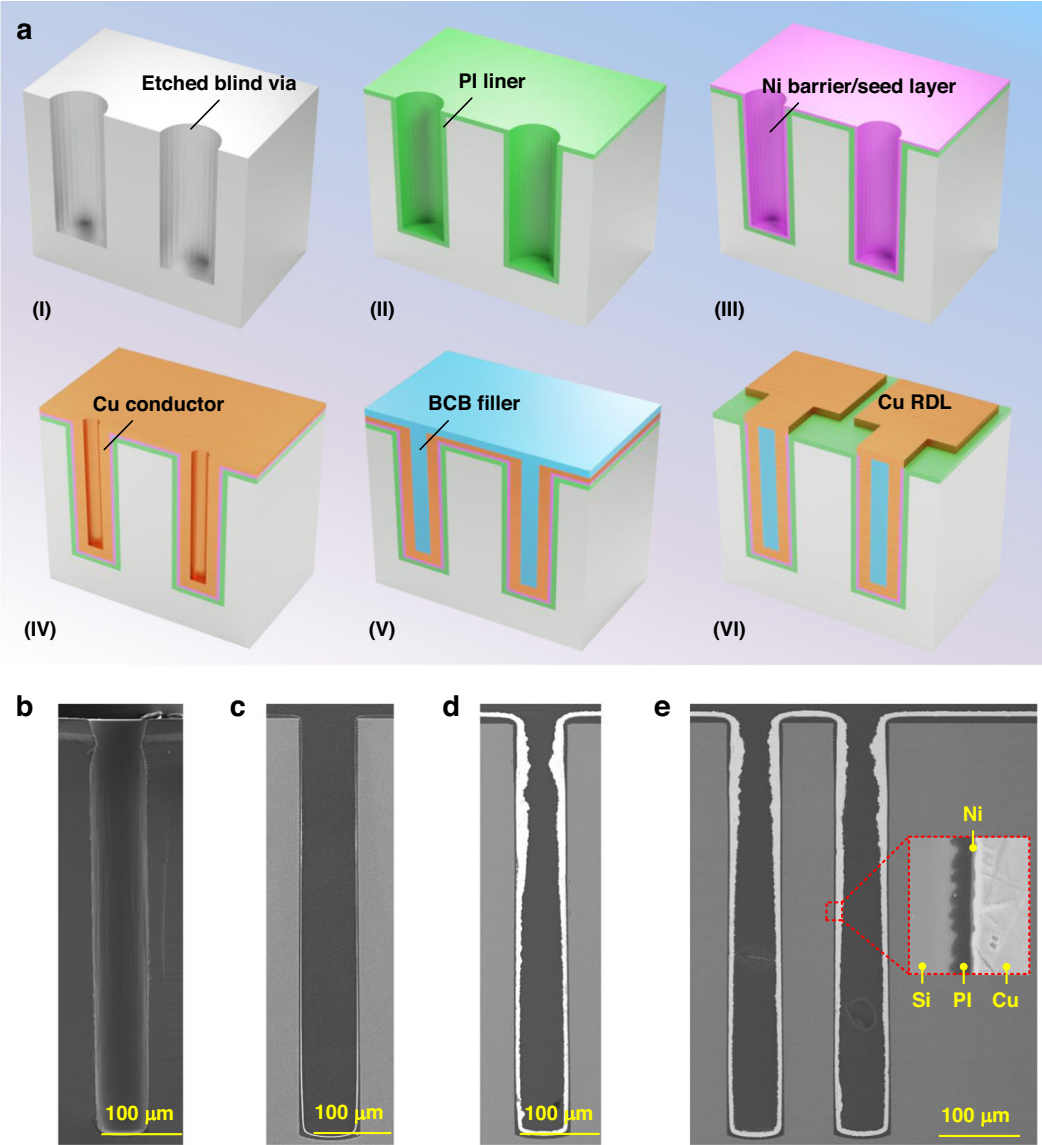
Complete fabrication flow for the ultra-deep TSV technique and the step-by-step fabrication results **a** Proposed fabrication flow: (I) etching of ultra-deep blind vias by DRIE, (II) deposition of PI liner by the vacuum-assisted spin-coating technique, (III) formation of Ni barrier/seed layer by the proposed dual catalysis-based ELP technique, (IV) electroplating of Cu annular conductors, (V) filling of BCB into the vias under vacuum treatment, and (VI) fabrication of RDLs. **b**–**e** SEM images of the fabrication results after the formation of PI liners, Ni barrier/seed layers, Cu annular conductors, and BCB fillers, respectively. Notably, the fillers in **c** and **d** are resin that is necessary for metallographic polishing, and the filler in **e** is BCB

Enabled by the proposed dual catalysis-based ELP technique, a cost-effective fabrication flow for ultra-deep TSVs was achieved, as summarized in Fig. 6a. The detailed process flow was as follows. First, deep blind vias were formed using DRIE (Fig. 6a(I)). A conformal PI liner was subsequently deposited by the vacuum-assisted spin coating technique featuring cost-effectiveness and a low temperature^39,40^, as shown in Fig. 6a(II). Afterward, a continuous Ni layer was deposited through the proposed dual catalysis-based ELP technique, serving as both a barrier layer and a seed layer (Fig. 6a(III)). Next, the electroplating of Cu was carried out, resulting in annular conductors, as shown in Fig. 6a(IV). BCB was then refilled into the vias under vacuum treatment conditions to prevent Cu oxidation, as shown in Fig. 6a(V). Finally, Cu redistribution layers (RDLs) were fabricated after removing the BCB overburden layer (Fig. 6a(VI)).

Figure 6b shows the SEM image of the deposited PI liner, and the magnified views are given in Fig. S6, revealing a continuous and conformal PI layer that can be distinctly observed along the sidewall of the entire via. The images show that the scallops induced by the Bosch etching process are effectively covered by the PI layer, presenting a smooth interface for subsequent ELP. This characteristic is beneficial for ensuring the continuity of the Ni layer and enhancing the reliability of the TSV structure.

By utilizing the continuous Ni layer fabricated by the dual catalysis-based ELP (Fig. 6c), annular Cu conductors were deposited by electroplating, as shown in Fig. 6d, where the sample was filled with resin and polished for a better demonstration. Following conductor formation, the central hollow vias were filled with BCB by the vacuum-assisted filling method, as shown in Fig. 6e, highlighting the successful fabrication of an ultra-deep TSV featuring a diameter of 70 μm and a depth of 530 μm. The formation of the annular Cu conductor verified the high-quality deposition of the Ni layer. Figures S7a, b, and c present the EDS scanning results of O, Ni, and Cu for a single TSV in Fig. 6e, corresponding to the PI liners, Ni barrier/seed layers, and annular Cu conductors, respectively. As shown in the inset of Fig. 6e, the functional layers could be clearly observed with a robust combination.

**Fig. 7:**
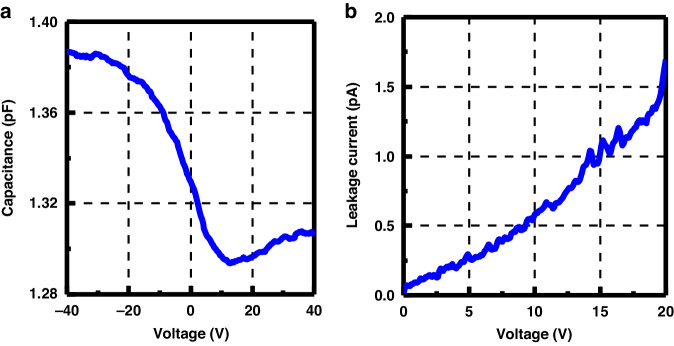
Electrical characterizations of the fabricated ultra-deep TSVs **a** C–V curve and **b** leakage current curve for a single TSV

Furthermore, the parasitic capacitance and leakage current of a single TSV were measured at room temperature. As shown in Fig. 7a, the capacitance within the depletion region was approximately 1.3 pF with a capacitance density of approximately 1.11 nF/cm^2^. This low capacitance density was attributed to the low *k* value (~2.9) of the PI and the substantial liner thickness. Additionally, as shown in Fig. 7b, the leakage current was as low as 1.7 pA at 20 V, proving the robust integrity and effective diffusion barrier capability of the Ni layer. The measured results were in accordance with similar TSVs^23,32^, and the minimal parasitic capacitance and leakage current values indicated the commendable electrical characteristics of the fabricated ultra-deep TSVs.

Notably, the TSVs demonstrated in this work had relatively large via diameters. ELP is a wet process that relies on a series of interfacial chemical reactions in a specific order. In addition, the plating rate at the via bottom decreased quickly with decreasing via diameter. Fortunately, due to the additional catalytic step involved in an alkaline environment, the proposed dual catalysis-based ELP technique could provide additional Pd catalysts at the via bottom. With further optimization, this technique has the potential to be applied to relatively small TSVs and to achieve challenging aspect ratios, which we will explore in the near future.

### Prospective application frameworks in 3D integration and packaging

**Fig. 8:**
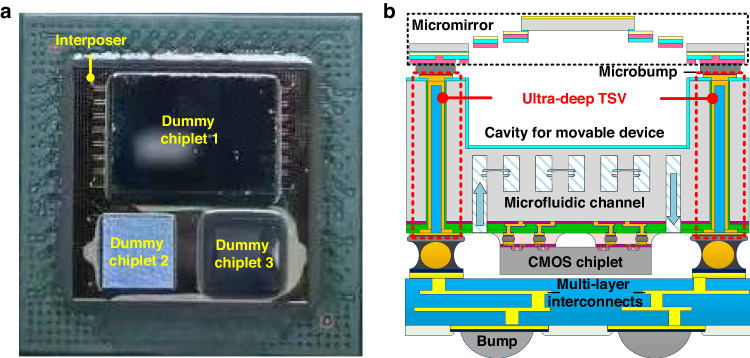
Application frameworks in microsystems **a** Optical image of a fabricated integrated system containing three dummy chiplets stacked on a TSV-embedded interposer. **b** Illustration of a microsystem exemplifying the need for ultra-deep TSVs. The ultra-deep TSVs play a pivotal role in facilitating electrical interconnections between the MEMS micromirror and CMOS chiplet within this complex integration architecture

Figure 8a shows a fabricated integrated system in which three dummy chiplets were stacked on a Si interposer containing TSVs. Moreover, Fig. 8b shows a representative diagram of a complicated heterogeneous integrated microsystem encompassing MEMS micromirrors, CMOS chiplets, and microfluidic channels, among other components. In this microsystem, a robust substrate with ultra-deep TSVs was considered essential for ensuring mechanical reliability. The TSV structure achieved through the method presented in this work was in agreement with the requirements of such integration architecture. With photonic devices such as micromirrors catering to optical functionalities, CMOS chiplets providing electrical capabilities, and microfluidic channels aiding in heat dissipation, this interposer featuring ultra-deep TSVs demonstrates significant potential for applications in optical, electronic, and thermodynamic heterogeneous integration systems and in the copackaging of multiple chiplets.

## Conclusions

In summary, continuous and dense Ni barrier/seed layers in ultra-deep TSVs were successfully fabricated by performing an innovative dual catalysis-based ELP technique. The additional catalytic step in an alkaline environment hydrolyzed the imide structure of the PI surface and formed the amide structure. Then, the exposed -COO^−^ groups attracted Pd^2+^ ions through electrostatic adsorption, resulting in the presence of additional Pd catalysts and a relatively dense Ni layer, as demonstrated by SEM and XPS. Notably, the proposed fabrication flow for ultra-deep TSV technique was characterized by its low cost and low temperature, omitting the high-cost steps like ALD. This process was all-wet besides the initial DRIE step. By leveraging the advantages of a low-*k* PI liner and a dense Ni barrier, the electrical properties of the fabricated TSVs exhibited favorable attributes, including low parasitic capacitance and leakage current values. This cost-effective and practical fabrication scheme presents a feasible solution for achieving high-performance ultra-deep TSVs, which are considered essential for the demands of heterogeneous multi-chiplet integration and packaging.

### Supplementary information

Supplemental Material
