# Supplementary material for: An ultra-deep TSV technique enabled by the dual catalysis-based electroless plating of combined barrier and seed layers
Source: Microsyst Nanoeng. 2024 Jun 11;10:76. doi: 10.1038/s41378-024-00713-5 (PMC11164994; doi:10.1038/s41378-024-00713-5)
Supplement: Supplementary file 1 — Supplemental Material [file 41378_2024_713_MOESM1_ESM.docx]

**Supplementary Information**

**An Ultra-Deep TSV Technique Enabled by the Dual Catalysis-Based Electroless Plating of Combined Barrier and Seed Layers**

**Yuwen Su ^1,2^, Yingtao Ding ^1^, Lei Xiao ^1^, Ziyue Zhang ^1,2 *^, Yangyang Yan ^2^, Zhifang Liu ^1^, Zhiming Chen ^1^, and Huikai Xie ^1,2 *^**

^1^School of Integrated Circuits and Electronics, Beijing Institute of Technology, Beijing 100081, China.

^2^Chongqing Institute of Microelectronics and Microsystems, Beijing Institute of Technology, Chongqing 400030, China.

*zyzhang@bit.edu.cn; hk.xie@bit.edu.cn


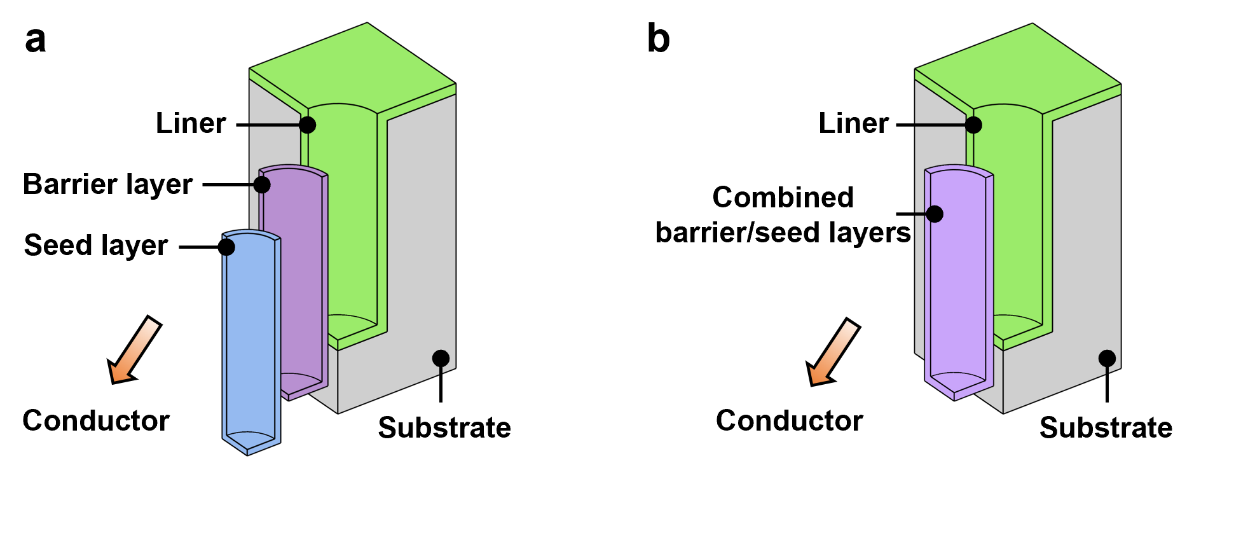


**Fig. S1 Schematics of the functional layers in typical TSV structures.** **a** A conventional TSV configuration where the functional layers contain a liner, a barrier layer, and a seed layer. **b** A novel TSV configuration where the barrier and seed layers are combined into a single layer.


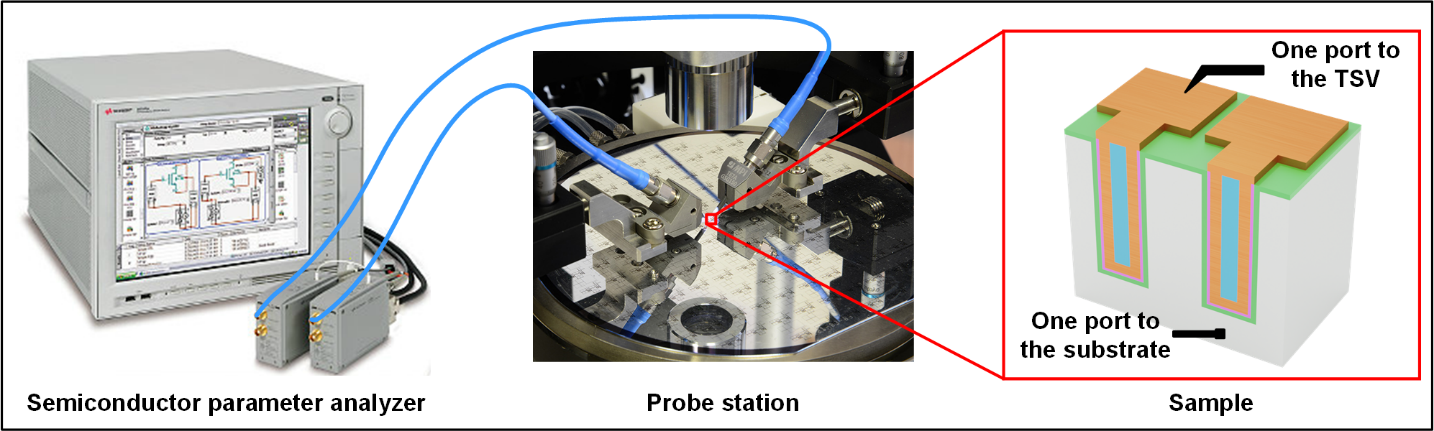


**Fig. S2 Schematic illustration of the test setup for electrical measurements.** Keysight B1500A semiconductor parameter analyzer was used to generate the test voltage, and the signal was transmitted to the probe station.


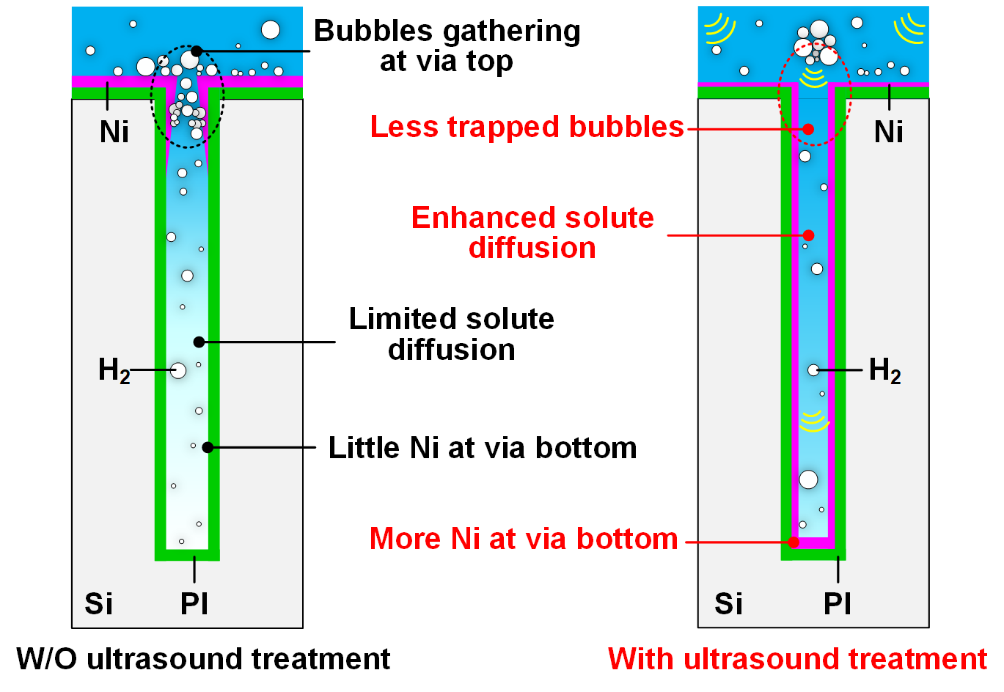


**Fig. S3** **Schematics of the effects of the ultrasound treatment on the Ni deposition.** The ultrasound treatment could enhance the escape of trapped bubbles and facilitate solute diffusion, thereby influencing the deposition of Ni.


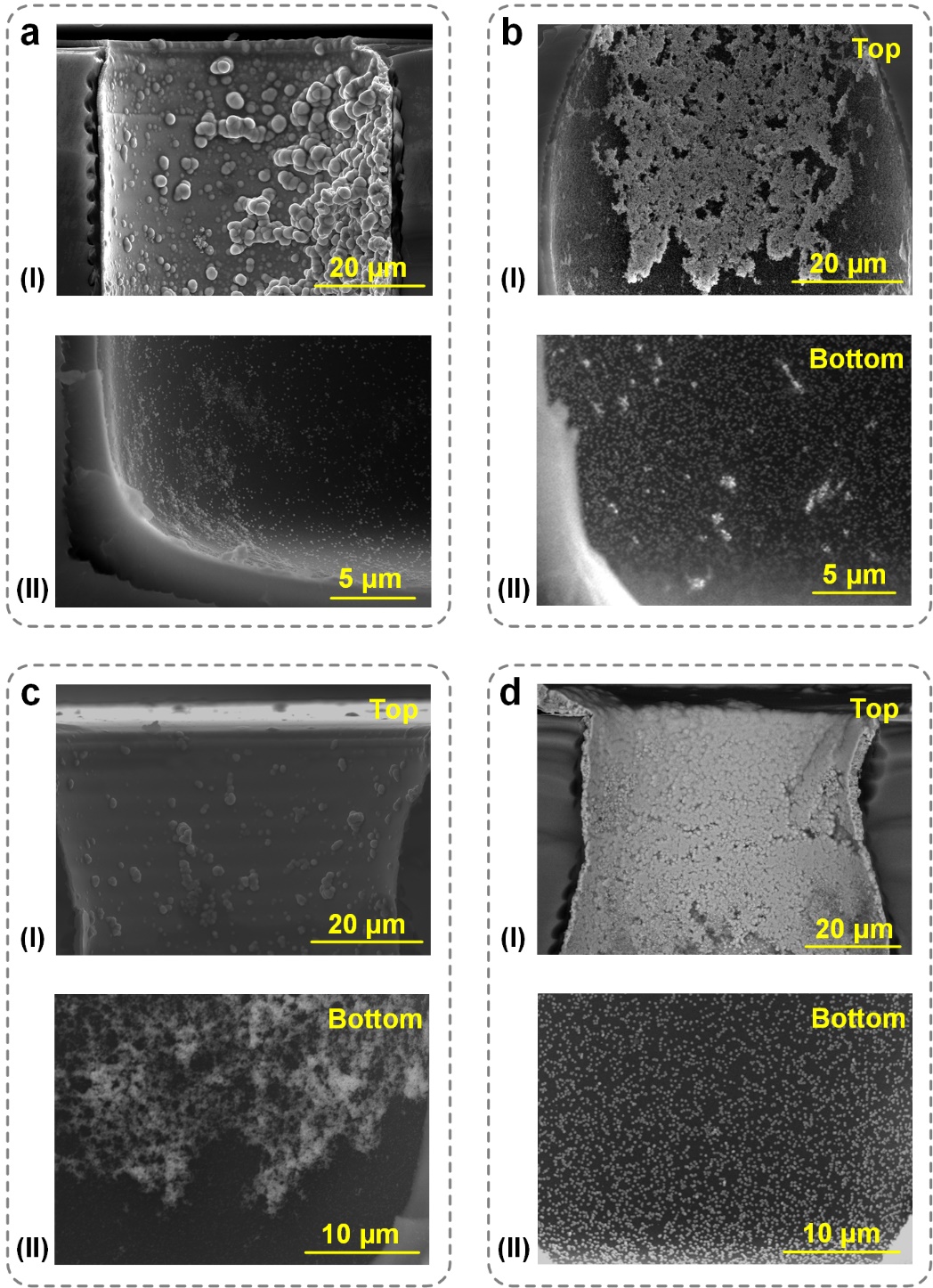


**Fig. S4** **SEM images of the Ni ELP results under ultrasound treatments with different ultrasound powers and pulse duty cycles. a** 100 W, 1 s/2 mins. **b** 200 W, 1 s/2 mins. **c** 300 W, 1 s/2 mins. **d** 300 W, 1 s/1 min.


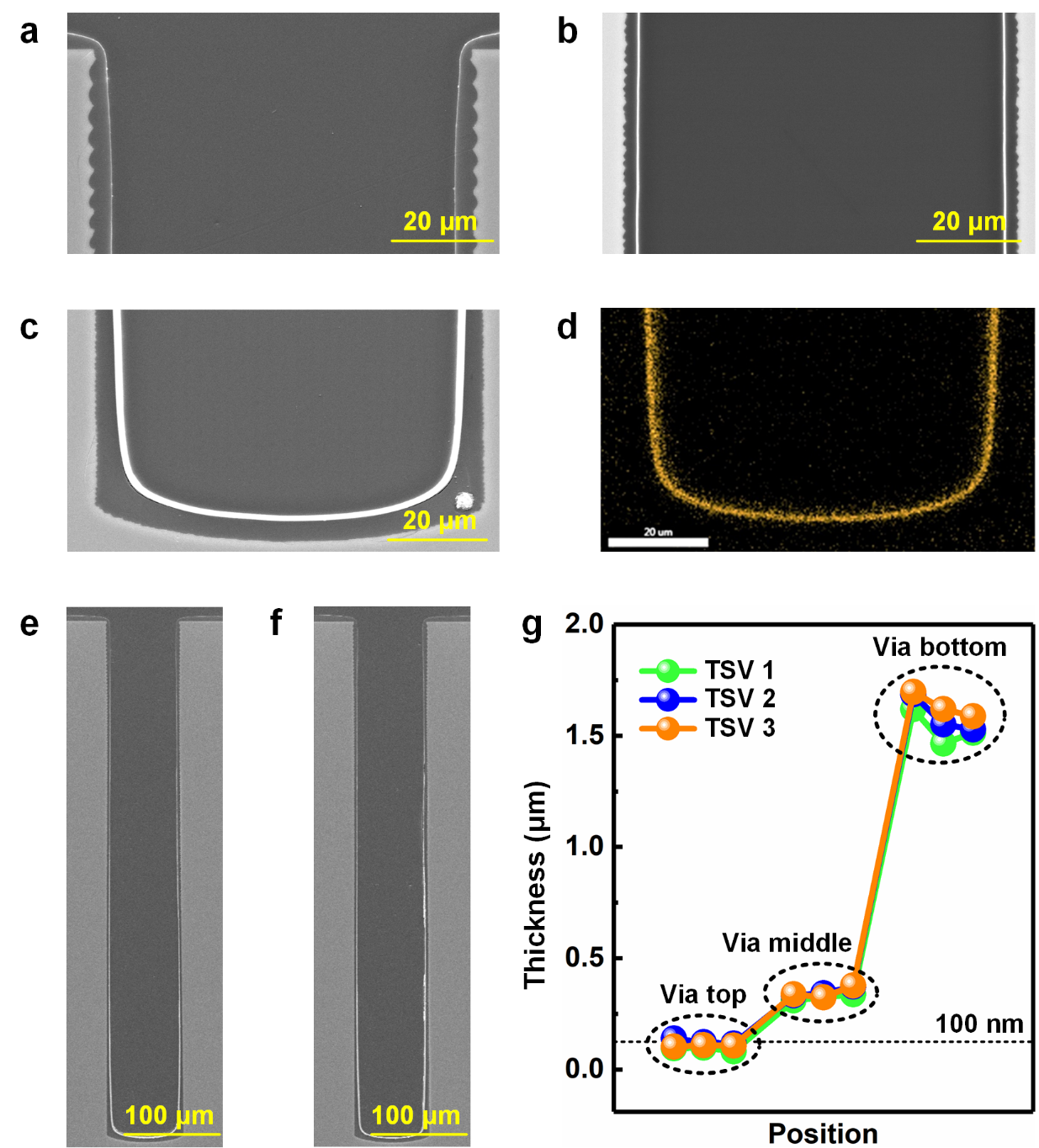


**Fig. S5 SEM images and thickness curves of the Ni ELP results in Case C.** **a-c** The enlarged via top, middle, and bottom of the TSV in **Fig. 3e**. **d** The EDS mapping result of Ni in **c**, proving the continuity of the Ni layer even at deep via bottom. **e** TSV located at the center of the wafer. **f** TSV located at the edge of the wafer. **g** Thickness curves of the Ni layers in the TSVs in **e** (TSV 1), **Fig. 3e** (TSV 2), and **f** (TSV 3).


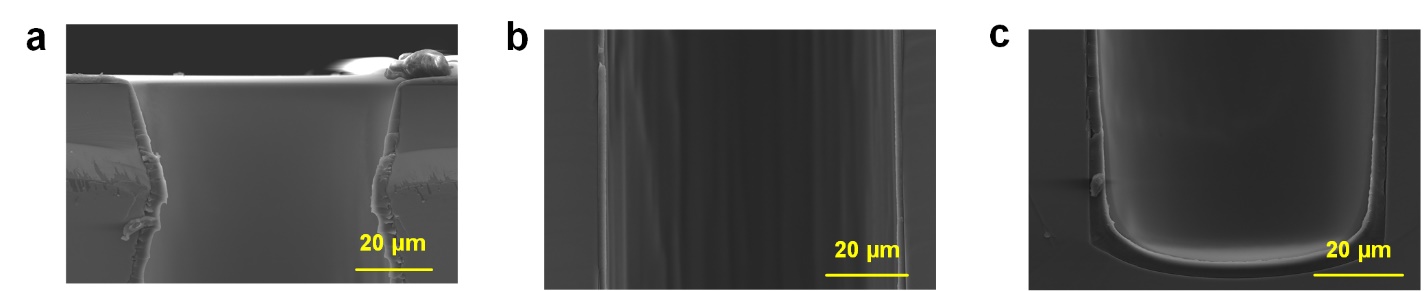


**Fig. S6 SEM images of the deposited PI liner. a** Via top. **b** Via middle. **c** Via bottom. It can be seen from the enlarged images that the PI layer was continuous and conformal with a relatively smooth surface.


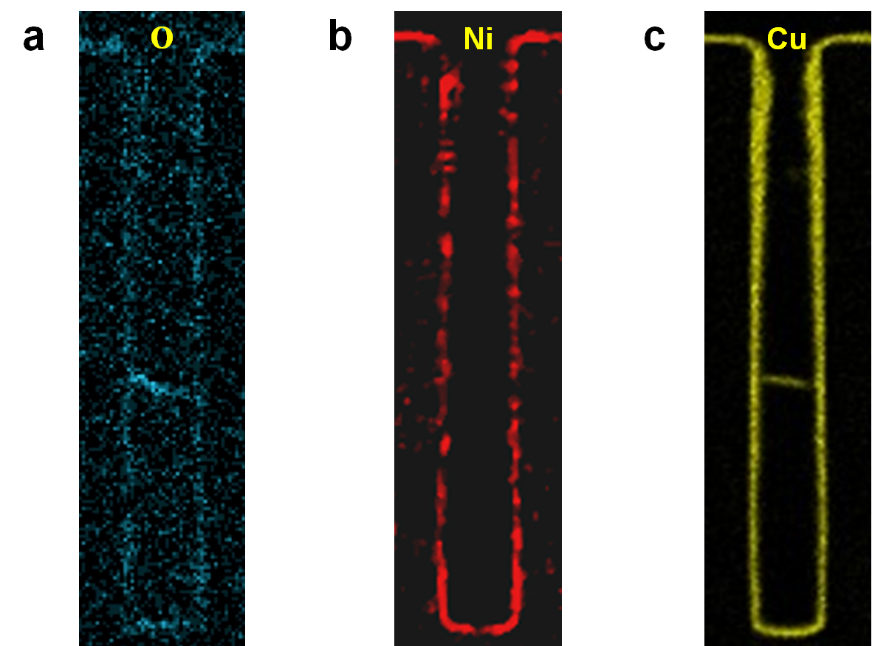


**Fig. S7 EDS mapping results of different elements for a single TSV. a** O. **b** Ni. **c** Cu.
